# Conserved miR156 Mediates Phase-Specific Coordination Between Cotyledon Morphogenesis and Embryo Dormancy During Somatic Embryogenesis in *Larix kaempferi*

**DOI:** 10.3390/ijms26178206

**Published:** 2025-08-23

**Authors:** Xin Li, Yuqin Huang, Wenhua Yang, Liwang Qi, Lifeng Zhang, Chenghao Li

**Affiliations:** 1 State Key Laboratory of Tree Genetics and Breeding, Northeast Forestry University, Harbin 150040, China; lx000628@163.com; 2 State Key Laboratory of Tree Genetics and Breeding, Key Laboratory of Tree Breeding and Cultivation of the National Forestry and Grassland Administration, Research Institute of Forestry, Chinese Academy of Forestry, Beijing 100091, China; huangyuqin0822@163.com (Y.H.); yangwh@caf.ac.cn (W.Y.); lwqi@caf.ac.cn (L.Q.)

**Keywords:** *MIR156*, *Larix kaempferi*, somatic embryogenesis, *Arabidopsis thaliana*

## Abstract

The miR156 family, crucial for phase transition and stress responses in plants, remains functionally uncharacterized in the ecologically and commercially important gymnosperm *Larix kaempferi*. This study systematically investigated *L. kaempferi* miR156 through phylogenetic analysis, structural prediction, expression profiling during somatic embryogenesis, and heterologous functional validation in *Arabidopsis*. Four *MIR156* family members (*LkMIR156*s) were identified in *Larix kaempferi*, each with a characteristic stem-loop structure and highly conserved mature sequences. Computational predictions indicated that these *LkMIR156*s target four *LkSPL* family genes (*LkSPL1*, *LkSPL2*, *LkSPL3*, and *LkSPL9*). qRT-PCR analysis showed that mature LkmiR156s expression remained relatively low during early embryonic development but was significantly upregulated at the cotyledonary stage (21–42 days). Precursor transcript levels peaked earlier (around 28 days) than those of the mature LkmiR156, which remained highly expressed throughout cotyledonary embryo development. This sustained high expression coincided with cotyledon morphogenesis and embryonic dormancy. Functional validation via heterologous overexpression of *LkMIR156b1* in *Arabidopsis* resulted in increased rosette leaf numbers (42.86% ± 6.19%) and individual leaf area (54.90% ± 6.86%), phenotypically consistent with the established role of miR156 in growth regulation. This study reveals the temporal expression dynamics of LkmiR156s during *L. kaempferi* somatic embryogenesis and its coordinated expression patterns with cotyledon development and embryonic dormancy. The functional conservation of the miR156-*SPL* module was confirmed in a model plant, providing key molecular insights into the developmental regulatory network of conifers. These findings offer potential strategies for optimizing somatic embryogenesis techniques in conifer species.

## 1. Introduction

MicroRNAs (miRNAs) are ubiquitous, single-stranded non-coding RNAs, typically 21–24 nucleotides (nt) in length, found across eukaryotes. They function primarily in the transcriptional and post-transcriptional regulation of gene expression by negatively controlling target mRNAs. Through base-pairing with complementary mRNA sequences, miRNAs guide silencing complexes to either degrade the transcript or inhibit its translation [1]. In plants, miRNAs modulate diverse biological processes, including growth and development, responses to biotic and abiotic stresses, and metabolite synthesis [2]. To date, thousands of miRNAs have been annotated in plants and animals. Among these, miR156 stands out as a highly conserved plant miRNA regulator. It governs flowering initiation and mediates the vegetative-to-reproductive phase transition in diverse plant species, encompassing model species (*Arabidopsis thaliana*), crops (*Zea mays*, *Oryza sativa*, *Glycine max*), and woody species, such as poplar [3]. Beyond regulating phase transitions, *MIR156* influences three key developmental aspects: (1) organ morphology, particularly via phyllotaxy regulation [4]; (2) metabolic pathways by modulating metabolite synthesis; and (3) hormonal signaling, notably involving gibberellin [5]. Despite extensive functional characterization in angiosperms, the roles of *MIR156* in gymnosperm plants remain largely unexplored.

*MIR156* targets members of the SQUAMOSA promoter-binding protein-like (*SPL*) gene family in plants. This large family encodes plant-specific transcription factors characterized by a highly conserved SBP DNA-binding domain [6]. In *Arabidopsis thaliana*, the *SPL* family comprises 17 members, 11 of which are subject to miRNA-mediated post-transcriptional regulation by miR156 [7]. Functional analyses demonstrate that *SPL9* and *SPL15* regulate developmental phase transitions, with *SPL9* overexpression accelerating the shift to reproductive growth [4,8]. Additionally, *SPL2*, *SPL10*, and *SPL11* play pivotal roles during embryogenesis [7]. Collectively, the miR156-*SPL* module constitutes a critical regulatory nexus governing plant development [9]. Recent studies have increasingly focused on this regulatory network due to its multifaceted roles in developmental processes, metabolic regulation, and abiotic stress responses [6,10,11,12]. Accumulating evidence confirms that this module serves as a central hub coordinating plant growth and development.

*Larix kaempferi* is one of China’s most important fast-growing plantation tree species. It is widely distributed across Northeast China, Japan, and Europe [13], playing a crucial role in both timber production and ecological construction. Somatic embryogenesis (SE) technology is a vital tool for large-scale clonal propagation of elite conifer varieties or genotypes with desirable traits. This technique has been applied in larch species for over 30 years [14] and is recognized as an ideal system for fundamental research on gymnosperm development and its regulatory mechanisms [13]. Numerous conserved miRNAs have been identified in *L. kaempferi* somatic embryos [15]. Among these, miR156 exhibits dynamic expression patterns across different developmental stages, suggesting its potential regulatory role in SE development. Our previous studies indicate that the miR156-*LkSPL9* module might participate in early embryonic patterning in *L. kaempferi* [13]. Furthermore, in *citrus*, the miR156-*SPL* module has been shown to play a critical role during the early SE induction stage by regulating starch accumulation in callus tissues [16]. Collectively, these findings indicate that the miR156-*SPL* regulatory module serves as a key regulator in SE processes.

The miR156 family is a highly conserved group of plant microRNAs that play crucial roles in regulating developmental timing, phase transition, and stress responses, yet its functional mechanisms in gymnosperms remain poorly understood. *Larix kaempferi* represents an ecologically and commercially important gymnosperm species, which lacks a comprehensive investigation into its miR156-mediated regulatory networks. This study aims to bridge this knowledge gap by: (i) elucidating evolutionary relationships through phylogenetic analysis, (ii) characterizing molecular features via secondary structure prediction, (iii) quantifying dynamic expression patterns of precursors/mature miRNAs during somatic embryogenesis, and (iv) validating biological functions through heterologous overexpression in *Arabidopsis.* These findings provide a foundation for future functional studies of *MIR156* in *L. kaempferi*.

## 2. Results

### 2.1. Cloning and Characterization of MIR156 in Larix kaempferi

To identify *MIR156* in *L. kaempferi*, predicted sequences were cloned using specifically designed primers (Supplementary Table S1). PCR-amplified fragments corresponded to expected sizes, as confirmed by agarose gel electrophoresis (Supplementary Figure S1). Finally, four *MIR156* sequences were obtained: One encoded the predicted mature miRNA LkmiR156a and was designated *LkMIR156a*; the other three encoded LkmiR156b and were named *LkMIR156b1*, *LkMIR156b2*, and *LkMIR156b3*, respectively.

### 2.2. Bioinformatic Analysis and Phylogenetic Reconstruction

**Figure 1:**
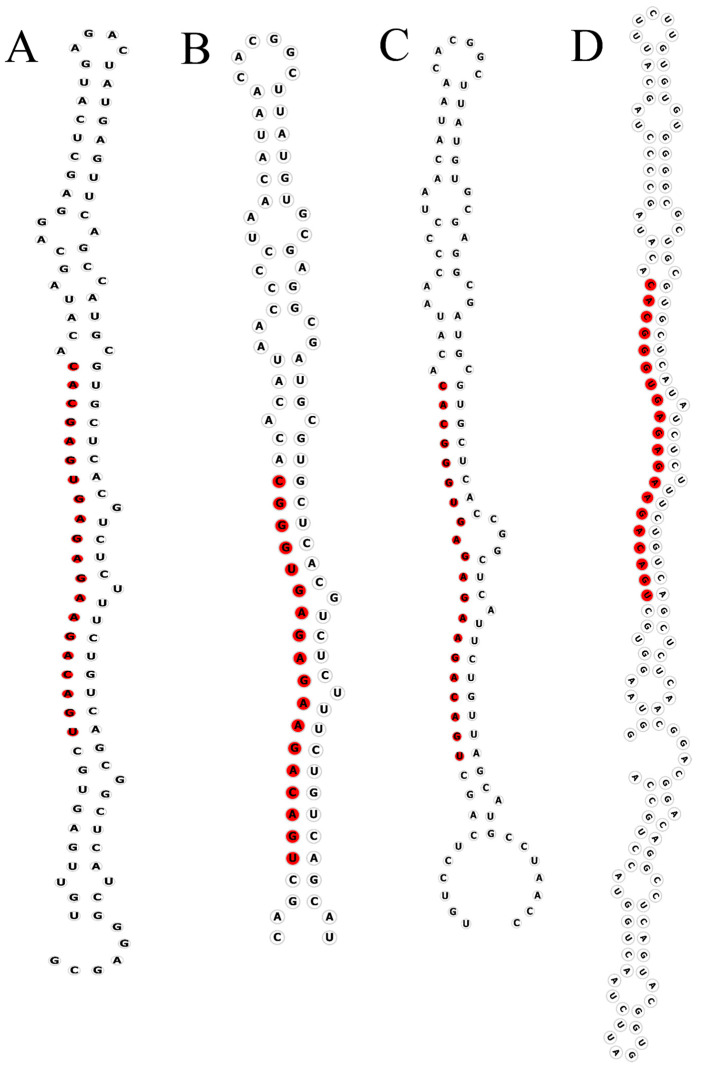
Secondary structures of Lkpre-miR156s. Mature miRNA sequences are highlighted in red. (**A**) Lkpre-miR156a; (**B**) Lkpre-miR156b1; (**C**) Lkpre-miR156b2; (**D**) Lkpre-miR156b3.

**Figure 2:**
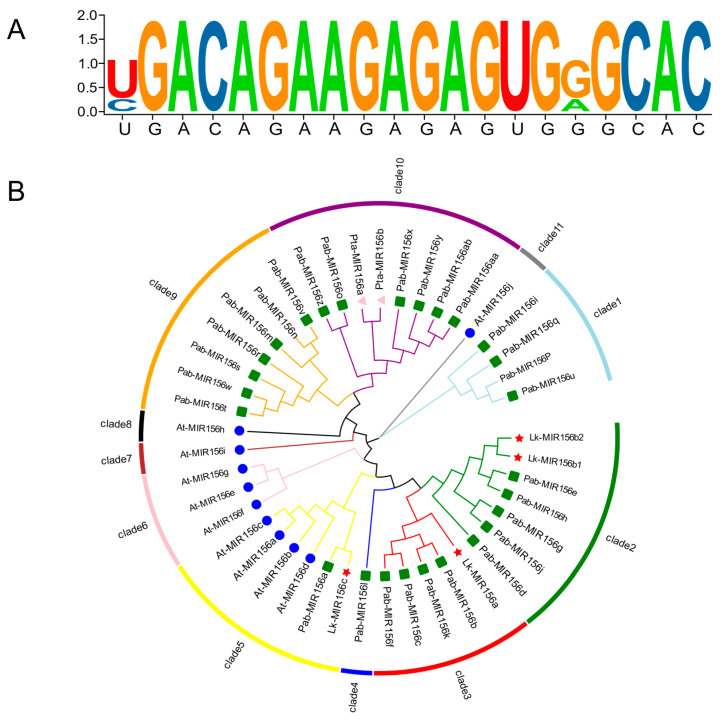
(**A**) Conservation analysis of mature miR156 sequences in nine plant species: *Citrus* spp., *Larix kaempferi*, *Picea* spp., *Pinus tabuliformis*, *Nicotiana tabacum*, *Triticum aestivum*, *Zea mays*, *Gossypium hirsutum,* and *Arachis hypogaea*. (**B**) Phylogenetic analysis of *MIR156* genes from four plant species. The dendrogram includes family members from *Arabidopsis thaliana* (At), *Larix kaempferi* (Lk), *Picea abies* (Pab), and *Pinus taeda* (Pta). Bootstrap resampling (*n* = 1000).

Secondary structures of cloned *L. kaempferi* pre-miR156s were predicted using RNA fold web server-univie.ac.at (http://rna.tbi.univie.ac.at/, accessed on 15 April 2025). (Figure 1). All family members formed canonical stem-loop structures with mature miRNA sequences embedded within the hairpin regions, consistent with typical miRNA architecture. However, structural variations were observed among different members, likely attributable to sequence divergence within the *MIR156* family. Alignment of mature sequences revealed that the four pre-miR156 members share two distinct mature forms with only a single nucleotide variation. Comparative analysis of mature miR156 sequences across nine plant species showed exceptionally high conservation (85% identity; Figure 2A). Phylogenetic reconstruction of 44 *MIR156* genes from multiple species resolved 11 distinct clades (Figure 2B). The four *LkMIR156* genes were distributed into three clades: one containing *LkMIR156b1* and *LkMIR156b2*, while *LkMIR156a* and *LkMIR156b3* each occupied separate clades. Notably, *LkMIR156* genes showed the highest sequence similarity with *PabMIR156*.

### 2.3. Identification of miR156 Target Genes in L. kaempferi and Arabidopsis Thaliana

**Figure 3:**
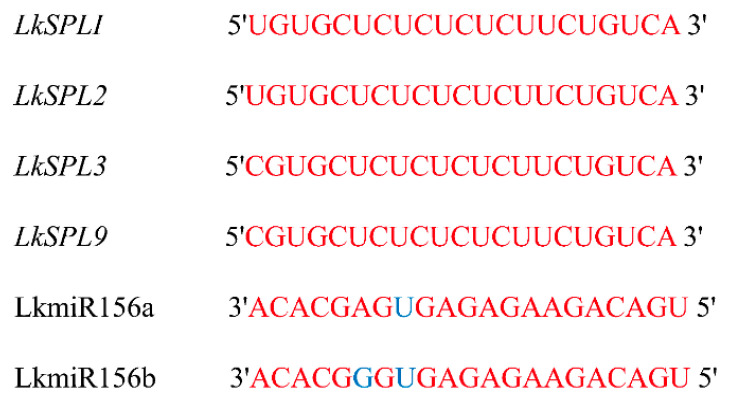
Sequence alignment of miR156s with *LkSPL1*, *LkSPL2*, *LkSPL3,* and *LkSPL9* transcripts (displaying the partial sequence fragments of *LkSPLs*).

Target prediction was done for LkmiR156s using the psRNATarget online server (http://www.zhaolab.org/psRNATarget/, accessed on 15 April 2025) with default parameters. The analysis identified *LkSPL1*, *LkSPL2*, *LkSPL3,* and *LkSPL*9 as predicted targets of miR156s (Figure 3). The analysis identified *AtSPL2*, *AtSPL6*, *AtSPL9*, *AtSPL10*, *AtSPL11*, *AtSPL13,* and *AtSPL15* as predicted targets of miR156 in *Arabidopsis thaliana* (Supplementary Figure S2).

### 2.4. Expression Profiling of Pre-miR156s in L. kaempferi SE

**Figure 4:**
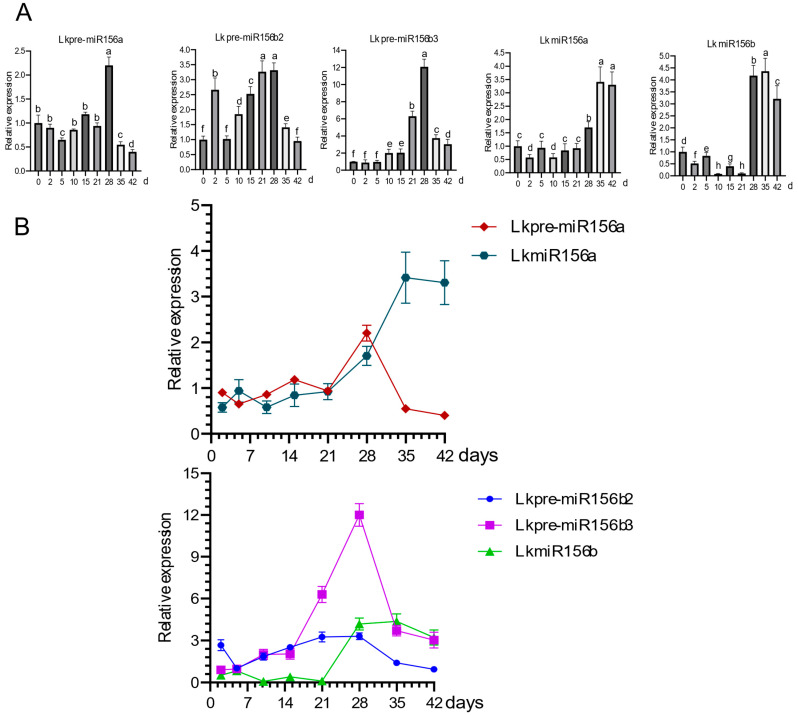
(**A**) Expression profiles of *Lkpre-miR156* and mature *LkmiR156* during *L. kaempferi* SE. Data are presented as mean ± SD (n = 3 biological replicates) and analyzed using SPSS 26.0 (*p* < 0.05). Letters are used to denote statistically significant differences among treatment groups. (**B**) Comparative expression dynamics of mature miR156 isoforms and corresponding precursors in *L. kaempferi* SE. Data are presented as mean ± SD (n = 3 biological replicates) and analyzed using SPSS 26.0 (*p* < 0.05).

The expression profiles of three pre-miR156 genes during SE were analyzed using quantitative reverse transcription PCR (qRT-PCR) (Figure 4A). Transcript levels showed a gradual increase during early development, which peaked at the early cotyledonary embryo stage (21–28 days after culture) and subsequently declined. This overall profile aligns with the pre-miR156b1 expression pattern reported in our previous study [17]. In *Arabidopsis*, AGO1 protein binds mature miRNAs and exhibits “RNA slicer” activity, cleaving target RNAs with perfect complementarity to small RNAs to mediate biological functions [18]. To elucidate the functional mechanism of the miR156-*SPL* module in SE, this study primarily analyzed the expression patterns of mature miR156s during SE development. qRT-PCR analysis revealed that two mature miR156 isoforms (*LkmiR156a* and *LkmiR156b*) with identical expression profiles during SE (Figure 4A). Both isoforms showed basal expression levels during the single-embryo stage but underwent significant transcriptional activation as development progressed through the cotyledonary stage (21–42 days), displaying a consistent upregulation trend. Comparative analysis of precursors and their mature forms during *L. kaempferi* SE revealed phase-shifted accumulation patterns. While three precursors (n = 3) reached maximal levels at 28 days before declining, mature miRNAs (n = 2) showed delayed peak expression at 35 days, followed by sustained accumulation through cotyledonary development (28–42 days), indicating precursors processing may represent a rate-limiting step in miR156 biogenesis during embryogenesis (Figure 4B).

### 2.5. Functional Analysis of LkMIR156b1 Through Heterologous Overexpression in Arabidopsis thaliana

**Figure 5:**
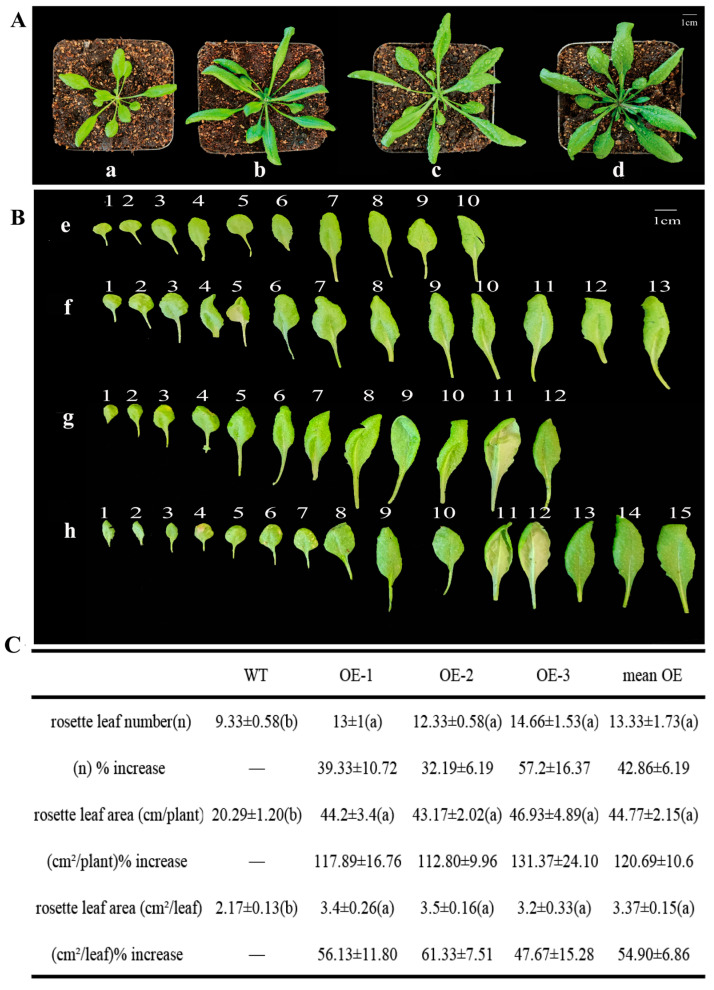
(**A**) Vegetative phenotypes of wild-type and *LkMIR156b1*-overexpressing *Arabidopsis* lines. (**a**) Wild-type (Col-0) plants. (**b**–**d**) Three independent *LkMIR156b1* overexpression lines (OE1–OE3) showing enhanced vegetative growth. Photographs taken 18 days after transplantation. (**B**) Rosette leaf morphology of wild-type and *LkMIR156b1*-overexpressing *Arabidopsis* lines. (**e**) Wild-type (Col-0) control. (**f**–**h**) Three independent *LkMIR156b1* overexpression lines (OE1–OE3) showing increased leaf size and number. (**C**) Phenotype data of wild-type and three lines of *LkMIR156b1*-overexpressing *Arabidopsis.* Data shown as mean ± standard deviation (SD). Data analyzed using SPSS 26.0, *p* < 0.05. Rosette leaf number (n): represents mean rosette leaf number; (n)% increase: represents percentage increase in mean rosette leaf number (%); rosette leaf area (cm^2^/plant): represents mean rosette leaf area per plant (cm^2^); (cm^2^/plant)% increase: represents percentage increase in mean rosette leaf area per plant (%); rosette leaf area (cm^2^/leaf): represents mean per rosette leaf area (cm^2^); (cm^2^/leaf)% increase: represents percentage increase in mean per rosette leaf area (%). Letters are used to denote statistically significant differences among treatment groups.

Zhang et al. [17] demonstrated that the pre-miR156b1 showed significantly higher expression levels compared to other precursors during somatic embryogenesis. To elucidate the biological function of *LkMIR156*, the *LkMIiR156b1* were selected for heterologous expression in *Arabidopsis.* Genomic PCR analysis of T1 transformants confirmed successful transgene integration, with amplified fragments matching the expected insert size (Supplementary Figure S3). Homozygous T3 lines were subsequently established for phenotypic analysis and qRT-PCR analysis. The OE-1 line was selected for qRT-PCR analysis, revealing higher mature miR156 expression in OE compared to WT, with six out of seven *SPL* target genes being downregulated (Supplementary Figure S4). Comparative analysis at 18 days post-transplantation revealed significant morphological differences between *LkMIR156b1*-overexpressing (OE) lines and wild-type (Col-0) controls (Figure 5A,B). Quantitative assessment of three independent transgenic lines (n = 5 plants per line) demonstrated: (1) 42.86% ± 6.19% (mean ± SD) increase in rosette leaf number; (2) 54.90% ± 6.86% expansion in individual leaf area (Figure 5C). These findings establish that *LkMIR156b1* overexpression promotes vegetative growth in *Arabidopsis*, supporting the evolutionarily conserved role of miR156 in regulating plant development.

## 3. Discussion

### 3.1. Evolutionarily Conserved Roles of miR156 in Plant Development

In this study, four *LkMIR156* family members were successfully cloned, which collectively encode two mature miR156 sequences (designated as miR156a and miR156b). Comparative sequence analysis of these mature miR156 variants across nine phylogenetically diverse plant species demonstrated 85% sequence identity, highlighting the extraordinary evolutionary conservation of miR156 in plants. This finding is consistent with previous extensive studies demonstrating that miR156 serves as a key regulator governing the phase transition from vegetative growth to reproductive development, primarily through its modulation of *SPL* gene family expression [6,10,11].

In *citrus*, miR156 has been demonstrated to regulate somatic embryogenic competence through its modulation of starch metabolism in callus cells [16]. Similarly, in rice, the developmental decline of miR156 levels facilitates reproductive transition by upregulating *OsSPL14* expression, resulting in accelerated reproductive growth and enhanced panicle branching [19]. The model plant *Arabidopsis* exhibits an age-dependent decrease in miR156 expression, which releases its repressive effect on *SPL9* and *SPL15* to initiate flowering [9,10]. This regulatory mechanism appears conserved in *Lycium ruthenicum*, where miR156-mediated suppression of *LrSPL* contributes to delayed flowering time [20].

Our qRT-PCR analysis revealed distinct spatiotemporal expression patterns of LkmiR156 during larch SE. The transcript levels remained relatively low during the single embryo stage but showed pronounced upregulation at the cotyledonary embryo stage. Notably, this expression pattern displayed an inverse correlation with the previously characterized expression profile of its putative *SPL* target gene [13,21], where *SPL* transcripts gradually accumulated during the single embryo stage but dramatically decreased at the cotyledonary embryo stage. These findings strongly suggest that LkmiR156 participates in *L. kaempferi* SE through negative regulation of the *SPL* gene. In *Arabidopsis*, qRT-PCR analysis revealed that the expression level of LkmiR156 was significantly higher than in the WT. Concurrently, six out of its seven target genes (*AtSPLs*) exhibited marked downregulation. These results suggest that LkmiR156 likely promotes vegetative growth in *Arabidopsis* by suppressing *AtSPLs* expression, thereby delaying flowering and prolonging the vegetative phase. This regulatory mechanism leads to increased leaf size and number. Our findings align with previous observations in larch, where LkmiR156 regulates somatic embryogenesis by negatively regulating *LkSPLs*.

Supporting the evolutionary conservation of miR156 function, heterologous overexpression of *LkmiR156b1* in *Arabidopsis* generated transgenic plants exhibiting characteristic miR156-overexpression phenotypes, including enlarged leaves and increased rosette leaf numbers. Detailed phenotypic analysis revealed that miR156 likely prolongs the juvenile phase through *SPL* suppression, which in turn enhances lateral meristem activity to promote rosette leaf production. Furthermore, the extended cell division and expansion phases mediated by miR156 account for the observed leaf size enlargement.

Notably, while the miR156 family demonstrates conserved regulatory functions across plant species, its target *SPL* genes exhibit significant quantitative variation among different species. Among the 17 *SPL* genes in *Arabidopsis thaliana*, 11 are targeted by miR156, whereas our preliminary studies identified only four out of 12 *SPL* genes are miR156-regulated in *L. kaempferi*. This striking divergence likely reflects adaptation to species-specific life history strategies: *Arabidopsis*, as a short-lived model plant completing its life cycle within 4–6 weeks, requires coordinated control through multiple *SPL* genes to ensure rapid developmental transitions. In contrast, the long-lived *L. kaempferi* (with a lifespan exceeding centuries) appears to have evolved a more selective regulatory strategy, depending on precise modulation of a core set of *SPL* genes to orchestrate its prolonged developmental program. These comparative findings offer new perspectives on how the miR156-*SPL* module has evolutionarily adapted to accommodate contrasting life history strategies in plants.

### 3.2. Stage-Specific Regulation of miR156 Biogenesis During Larch SE

Our Systematic expression analysis revealed distinct temporal patterns between miR156 precursors and mature forms during larch SE. Three precursor transcripts displayed progressive upregulation, peaking at 28 days (cotyledonary embryo stage initiation), while two mature miR156 isoforms maintained consistently low expression with progressive downregulation during the single embryo stage (0–21 days). This observed precursor-mature miRNA dissociation may result from: (1) limited DCL1 enzymatic activity, or (2) presence of processing inhibitors such as HYL1 [22]. The transition to cotyledonary embryogenesis (28–42 days) exhibited inverse dynamics—precursor levels declined while mature miRNA accumulated, suggesting either: (i) enhanced DCL1 complex efficiency, or (ii) activation of mature miRNA stabilization pathways. Notably, precursor peaks consistently preceded those of mature forms, reflecting the stepwise miRNA biogenesis process: transcription (pri-miRNA) → processing (pre-miRNA) → maturation (mature miRNA) [23]. This temporal decoupling implies sophisticated regulation through: (a) dynamic modulation of processing enzyme activity, and (b) stage-specific auxiliary factors that tune maturation rates according to cellular requirements [23]. In *Arabidopsis thaliana*, this canonical miRNA biogenesis process (pri-miRNA → mature miRNA) is also conserved. Pri-miRNAs are precisely processed into pre-miRNAs by the DCL1 complex [24,25]. The HASTY protein facilitates the nuclear export of pre-miRNAs [26], ultimately leading to the formation of miRNA duplexes in the cytoplasm. The guide strand (mature miRNA) is selectively incorporated into the RNA-induced silencing complex (RISC) through its association with AGO1 protein [18], while the passenger strand (miRNA strand) is degraded by the SDN nuclease [27], thereby completing the miRNA maturation process.

qRT-PCR analysis further revealed greater stability of mature forms compared to precursors (which showed significant downregulation after 28 days), possibly mediated by AGO1 (Argonaute 1)-dependent protection of mature miRNAs from degradation [28]. These results establish three key regulatory principles: (1) precursor stockpiling enables rapid mature miRNA production when needed; (2) differential stability controls prevent premature target suppression; and (3) the maturation bottleneck ensures developmental stage-appropriate miRNA activity.

Cross-species analysis of nine plant species revealed mature miR156 sequence conservation (85% identity) despite significant precursor sequence variation. This phenomenon parallels observations in rice, where conserved miRNAs (e.g., miR156/miR172) are encoded by multiple genomic loci yet yield identical mature sequences [29]. These findings highlight two evolutionary strategies: (1) functional redundancy through multiple precursor genes, and (2) strong selective pressure maintaining mature sequence fidelity despite precursor diversification.

### 3.3. Regulatory Functions of the miR156-SPL Module During SE in Larix kaempferi

Extensive studies have established that miRNAs serve as key temporal regulators of embryonic development through precise modulation of target gene expression [30,31]. Our findings reveal that during *L. kaempferi* SE, the miR156*-SPL* module forms a canonical negative feedback loop that orchestrates stage-specific developmental transitions. qRT-PCR analysis showed that significant upregulation of mature miR156a/b during cotyledonary embryo formation (21–35 days), coinciding with marked downregulation of target *SPL* transcripts [13,21]. These results parallel observations in *Arabidopsis* shoot development, where miR156-mediated *SPL* suppression delays juvenile-to-adult transition [10], confirming the evolutionary conservation of this regulatory mechanism in plants.

Previous studies have demonstrated that *LkmiR156b* exhibits significantly higher expression abundance compared to *LkmiR156a* during somatic embryogenesis. Therefore, we propose that *LkmiR156b* likely plays a predominant regulatory role in this developmental process. The mature miR156a showed a gradually increasing expression pattern throughout development, whereas miR156b exhibited significant downregulation at specific stages (e.g., 2 days, 10 days, 21 days). We propose the following potential explanations for this differential regulation: during the early single-embryo stage, following the transfer of embryogenic suspension masses (ESMs) to maturation medium, the hormonal profile undergoes significant alteration (with depletion of auxins and cytokinins). This hormonal shift likely accounts for the observed downregulation of *LkmiR156b* expression at 2 days. Notably, at 10 days post-transfer, *LkmiR156b* shows marked downregulation, while transcript levels of its regulatory targets *LaSPL2* and *LaSPL9* peak simultaneously [13,21]. At 21 days, during the cotyledonary embryo formation stage, mature miR156b expression was significantly downregulated, while its target gene *SPL1* expression peaked at this developmental phase. miR156 expression remains relatively low while *SPL* transcripts progressively accumulate [13,21], suggesting that limited precursor processing efficiency during this developmental window may lead to insufficient mature miR156 production, thereby permitting transient SPL upregulation to activate embryonic patterning genes. This regulatory strategy contrasts sharply with animal systems, where miRNAs typically accelerate developmental transitions [26], highlighting a unique plant-specific developmental paradigm. As embryogenesis progresses, elevated miR156 expression during cotyledonary formation effectively suppresses *SPL* to ensure proper organ differentiation and shoot apical meristem establishment. Intriguingly, during late cotyledonary stages (35–42 days), declining miR156 levels appear to facilitate embryo maturation through dual mechanisms: (1) gradual release of SPL suppression and (2) protection of key regulatory transcripts (e.g., LEC1/2) from miRNA-mediated degradation [32,33], thereby enabling the synthesis of late-accumulating proteins such as seed storage proteins [34].

Our findings establish that the miR156-SPL module orchestrates *L. kaempferi* SE through a sophisticated, phase-specific regulatory mechanism. This temporal control system operates via precisely coordinated expression dynamics: (1) permitting *SPL* accumulation during early embryogenesis to initiate patterning processes, (2) enforcing *SPL* suppression at intermediate stages to direct organogenesis, and (3) gradually releasing *SPL* inhibition during late phases to promote maturation events. These results significantly deepen our mechanistic understanding of SE in gymnosperms while providing compelling phylogenetic evidence for the evolutionarily conserved regulatory roles of plant miRNAs.

## 4. Materials and Methods

### 4.1. Plant Materials and Culture Conditions

Immature zygotic embryos of *L. kaempferi* were inoculated onto solid induction medium to initiate embryonal-suspensor masses (ESMs), which were subsequently subcultured every three weeks on solid proliferation medium [13]. A highly embryogenic cell line (C6) was selected and cultured on proliferation medium for 15 days before being transferred to differentiation medium to induce somatic embryo formation. Seven distinct developmental stages were sampled: Proembryogenic masses (proliferation phase), Early single embryos (1–5 days on maturation medium), Mid-stage single embryos (5–14 days), Late single embryos (15–21 days), Early cotyledonary embryos (21–28 days), Mid-stage cotyledonary embryos (28–35 days), and Late cotyledonary embryos (35–42 days).

*Arabidopsis thaliana* seeds were stratified at 4 °C for 48 h, surface-sterilized with 70% ethanol (30 s) followed by 0.1% sodium hypochlorite (NaClO) solution for 6 min, and rinsed thoroughly with sterile water (three times). Seeds were sown on 1/2MS medium and cultured in a growth chamber (16 h light/8 h dark cycle, 22 °C, 80% humidity). Seedlings at the two-true-leaf stage were transplanted into a greenhouse potting mix [v (peat-based substrate)/v (vermiculite) = 1:1] for further growth.

### 4.2. Identification and Cloning of MIR156s

The potential sequences of the *MIR156* family were retrieved from the National Center for Biotechnology Information (NCBI) genome database and aligned with the transcriptome database of *Larix kaempferi*. The mature miRNA positions were analyzed using the miRBase online tool http://www.mirbase.org/, accessed on 15 April 2025). The secondary structures of precursors were predicted using RNAfold web server-univie.ac.at (http://rna.tbi.univie.ac.at/, accessed on 15 April 2025).

To experimentally validate the predicted LkmiR156 candidates, primers were designed using DNAMAN 9.0 software. Total RNA was extracted from *Larix* SE-stage materials using the Total RNA Extraction Kit (ZOMANBIO, Beijing, China). Reverse transcription was conducted to generate cDNA templates, which served as amplification templates for PCR using sequence-specific primers. Amplified products were cloned into the pEASY vector (TransGen Biotech, Beijing, China) and transformed into Trans1-T1 competent cells (TransGen Biotech, Beijing, China). Transformed colonies were subjected to overnight culture at 37 °C, followed by liquid culture of single colonies. PCR verification and sequencing analysis were performed to confirm the cloned sequences. 

### 4.3. Bioinformatics Analysis and Phylogenetic Tree Construction

The stem-loop secondary structure of the *L. kaempferi* miR156 sequences was predicted using the online RNA fold web server-univie.ac.at (http://rna.tbi.univie.ac.at, accessed on 15 April 2025) The mature miR156 sequences from *Citrus*, *Larix kaempferi*, *Picea* spp., *Pinus tabuliformis*, *Nicotiana tabacum*, *Triticum aestivum*, *Zea mays*, *Gossypium hirsutum,* and *Arachis hypogaea* in the miRBase database (https://www.mirbase.org/, accessed on 15 April 2025). Sequence conservation of LkmiR156 mature sequences was performed using WEB Logo (http://weblogo.berkeley.edu/logo.cgi, accessed on 15 April 2025). Ten *MIR156* gene family sequences of *Arabidopsis thaliana* were obtained from the miRBase database (https://www.mirbase.org/, accessed on 15 April 2025), while two sequences from *Pinus taeda* and 28 sequences from *Picea abies* were retrieved from the miRBase database (https://www.mirbase.org/, accessed on 15 April 2025). Phylogenetic analysis of these sequences was conducted using MEGA 11 software. The neighbor-joining (NJ) method was employed with bootstrap analysis set to 1000 replicates.

### 4.4. Target Gene Prediction of miR156 in L. kaempferi

Bioinformatic prediction of miR156 target genes in *L. kaempferi* was performed using the psRNATarget platform (http://www.zhaolab.org/psRNATarget/, accessed on 15 April 2025).

### 4.5. Expression Analysis of Pre-miR156s During SE in L. kaempferi

Total RNA was extracted from samples using RNAiso Plus (TaKaRa, Dalian, China) supplemented with RNAisomate plant tissue RNA extraction aid (TaKaRa). For cDNA synthesis, 1 μg of total RNA was treated with PrimeScript™ RT Reagent Kit with gDNA Eraser (Perfect Real Time; TaKaRa) to eliminate genomic DNA contamination. Quantitative real-time PCR (qRT-PCR) was performed using SYBR Premix Ex Taq™ (TaKaRa) on a CFX96™ Real-Time PCR Detection System (Bio-Rad, Shanghai, China) to measure *Lkpre-miR156s* expression levels. All primer sequences used are provided in Supplementary Table S2. Relative gene expression was calculated using the 2^−ΔΔct^ method with LkEF1A1 (GenBank accession: JR153706) as the reference gene. Three biological replicates were included in the experiment (n = 3). Statistical analysis was conducted using SPSS 26.0 software, with one-way ANOVA employed to determine significant differences in expression levels among tissues (significance threshold set at *p* < 0.05).

### 4.6. Expression Analysis of Mature miR156 During SE in L. kaempferi

Total small RNA was isolated from nine developmental stages of somatic embryos using the MicroRNA Kit (ZOMANBIO, Beijing, China). First-strand cDNA was synthesized from small RNA using the miRcute Enhanced miRNA cDNA Synthesis Kit (TIANGEN, Beijing, China), followed by a 10-fold dilution for qRT-PCR analysis. The reverse primer was the universal primer supplied with the kit, while the forward primers were: miR156F1: 5′-GGCGTGACAGAAGAGAGTGAGCAC-3′, miR156F2: 5′-GAGCTGACAGAAGAGAGTGGGCACA-3′. Based on preliminary screening and validation, 5.8S rRNA was selected as the reference gene, with its forward primer sequence: 5′-GTCTGTCTGGGCGTCGCATAA-3′. Relative gene expression levels were calculated using the 2^−ΔΔct^ method. Three biological replicates were included in the experiment (n = 3). Statistical analysis was performed using SPSS 26.0 software, with one-way ANOVA employed to assess significant differences in expression levels among different developmental stages (significance threshold set at *p* < 0.05).

### 4.7. Transformation of Arabidopsis Thaliana

Total RNA was extracted from *Larix kaempferi* using the Trizol method. First-strand cDNA was synthesized from the RNA template using the PrimeScript™ II 1st Strand cDNA Synthesis Kit (TaKaRa, Beijing, China). Following vector construction principles, specific primers containing *Hin*d III and *Spe* I restriction sites were designed using DNAMAN software: Forward primer (F): 5′-CCCAAGCTTTTGTACTCAGCCGACAGAA-3′; Reverse primer (R): 5′-GGACTAGTCCTCTAGCGGTAAATCTCAA-3′. The miR156b1 was PCR-amplified using cDNA template and the designed primers. Amplification products were verified by 1% agarose gel electrophoresis, excised, and purified. The target fragment was cloned into the pEASY-Blunt vector (TransGen Biotech, Beijing, China) and transformed into competent cells for sequencing verification. Positive clones were cultured overnight at 37 °C (200 rpm) in LB medium containing kanamycin (50 mg/L). Plasmid DNA was extracted using a TaKaRa plasmid extraction kit (Dalian, China). For vector construction, both the miR156b1-containing plasmid and pSuper-1300 expression vector were digested with *Hin*d III/*Spe* I. Gel-purified fragments were ligated using T4 DNA ligase. Recombinant plasmids were verified by colony PCR before transformation into *Agrobacterium tumefaciens* GV3101 competent cells (TransGen Biotech, Beijing, China). Floral dip transformation was performed by immersing *Arabidopsis* inflorescences in *Agrobacterium* suspension for 20–30 s [35]. Post-infection, plants were maintained horizontally in high humidity under dark conditions for 18–24 h before returning to normal growth. T_0_ transgenic seeds were screened on 1/2MS medium containing hygromycin (50 mg/L), with resistant seedlings used for subsequent analysis.

### 4.8. Screening and Verification of Transgenic Arabidopsis Plants

The recombinant plasmid carrying the target fragment was transformed into wild-type (WT) *Arabidopsis thaliana* plants via the floral dip method (*Agrobacterium*-mediated transformation). T_0_ seeds were harvested, surface-sterilized, and plated on 1/2MS medium supplemented with hygromycin (50 mg/L) for selection. After approximately two weeks of cultivation, putative transgenic seedlings showing normal growth under hygromycin selection were identified as positive transformants, while non-transgenic plants exhibited severe growth inhibition and typically died within 10 days post-germination [36]. Hygromycin-resistant T_0_ plants were transferred to nutrient-rich soil and grown to maturity. T_1_ seeds were collected and subjected to secondary selection on hygromycin-supplemented medium. Genomic DNA was extracted from leaf tissues of surviving T_1_ plants and analyzed by PCR to verify transgene integration. Positive T_1_ plants were transplanted to soil and cultivated for three generations to obtain genetically stable T_3_ homozygous lines for subsequent phenotypic and molecular analyses.

### 4.9. Expression Analysis of LkmiR156 and AtSPLs in Transgenic Arabidopsis Plants

The methods and procedures followed those described in Section 4.5 and Section 4.6. The primers used for qRT-PCR are listed in Supplementary Table S3. Data are presented as mean ± SD (n = 3 biological replicates). Statistical significance was determined by Student’s *t*-test. (* *p* < 0.05, ** *p* < 0.01).

## 5. Conclusions

This study identified four *LkMIR156s* family members from *L. kaempferi*, all containing canonical stem-loop structures and encoding two mature variants (miR156a and miR156b) that potentially regulate four *LkSPL* transcription factors. Heterologous functional assays confirmed the evolutionary conservation of the miR156-*SPL* regulatory module in conifers. Temporal expression profiling revealed distinct accumulation patterns: precursor transcripts peaked earlier than their mature counterparts, while mature miR156s maintained sustained high expression during critical phases of cotyledonary embryo development and maturation. These expression dynamics correlate precisely with key morphogenetic events in larch SE, including cotyledon specification and embryonic dormancy establishment, strongly implicating LkmiR156s as key regulators of these processes. However, direct molecular evidence for the targeting relationship between LkmiR156s and *LkSPLs* remains to be established, and further investigation is needed to elucidate the downstream regulatory network of the miR156-*SPL* module. This study provides new insights into the developmental regulatory mechanisms of conifers and lays an important theoretical foundation for improving somatic embryogenesis techniques in conifers.

## Data Availability

All data in this study can be found in the manuscript or the Supplementary Materials.

## Supplementary Materials

The following supporting information can be downloaded at: https://www.mdpi.com/article/10.3390/ijms26178206/s1.
