# Supplementary material for: Conserved miR156 Mediates Phase-Specific Coordination Between Cotyledon Morphogenesis and Embryo Dormancy During Somatic Embryogenesis in Larix kaempferi
Source: Int J Mol Sci. 2025 Aug 23;26(17):8206. doi: 10.3390/ijms26178206 (PMC12427833; doi:10.3390/ijms26178206)
Supplement: Supplementary file 1 [file ijms-26-08206-s001.zip › ijms-3718983-supplementary.pdf]

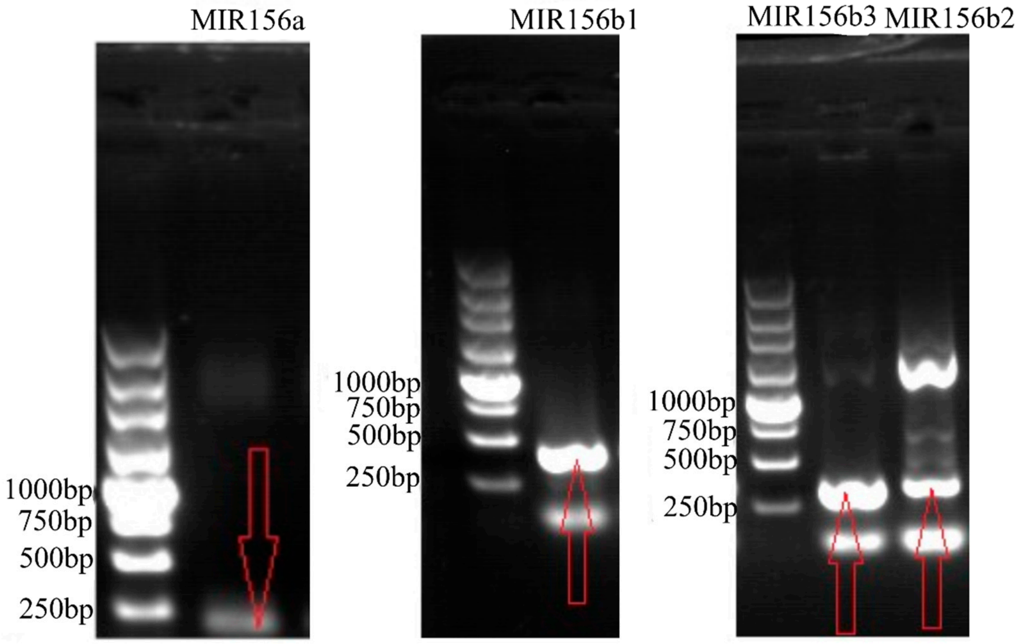

Supplementary Fig.S1. Analysis of *LkMIR156s* by agarose gel electrophoresis. The red arrow indicates the target fragment.

AtSPL2	**5‘GGUGCUCUCUCUUCUGUCA3’**
AtSPL6	**5‘CGUGCUCUCUCUUCUGUCA3’**
AtSPL9	**5‘UGUGCUCUCUCUUCUGUCA3’**
AtSPL10	**5‘AGUGCUCUCUCUUCUGUCA3’**
AtSPL11	**5‘CGUGCUCUCUCUUCUGUCA3’**
AtSPL13	**5‘UGUGCUCUCUCUUCUGUCA3’**
AtSPL15	**5‘UGUGCUCUCUCUUCUGUCA3’**
**LkmiR156b**	**5‘ACACGGGUGAGAGAAGACAGU3’**

Supplementary Fig.S2 Sequence alignment of miR156s with *AtSPLs* transcripts (displays the partial sequences fragments of *LkSPLs*).

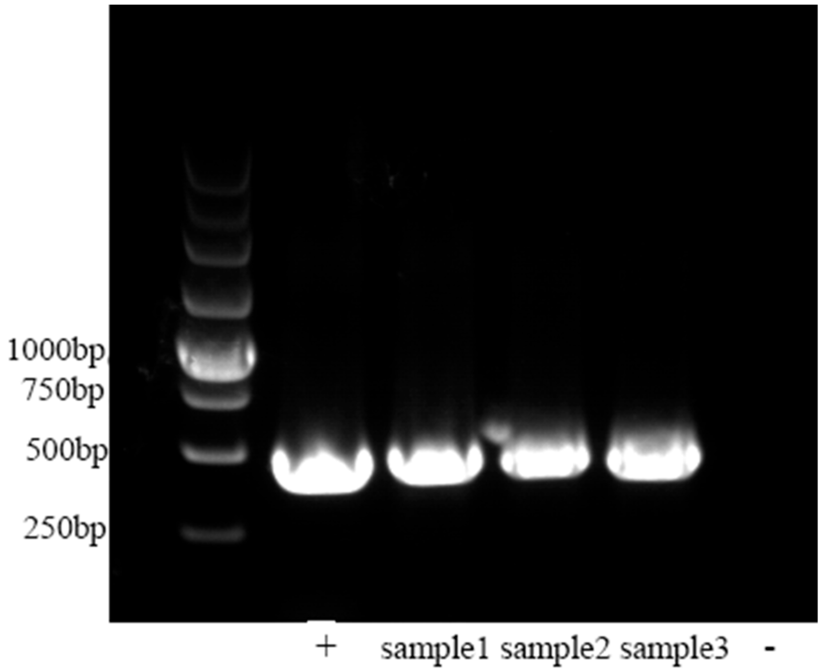

Supplementary Fig.S3. PCR-positive verification of three lines of *Lk~~mi~~MIR156b1*-overexpressing *Arabidopsis.*

" +":indicates positive; "–": indicates negative control; sample1: indicates OE-LkmiR156b1-1; sample2: indicates OE-LkmiR156b1-2; sample3: indicates OE-LkmiR156b1-3.

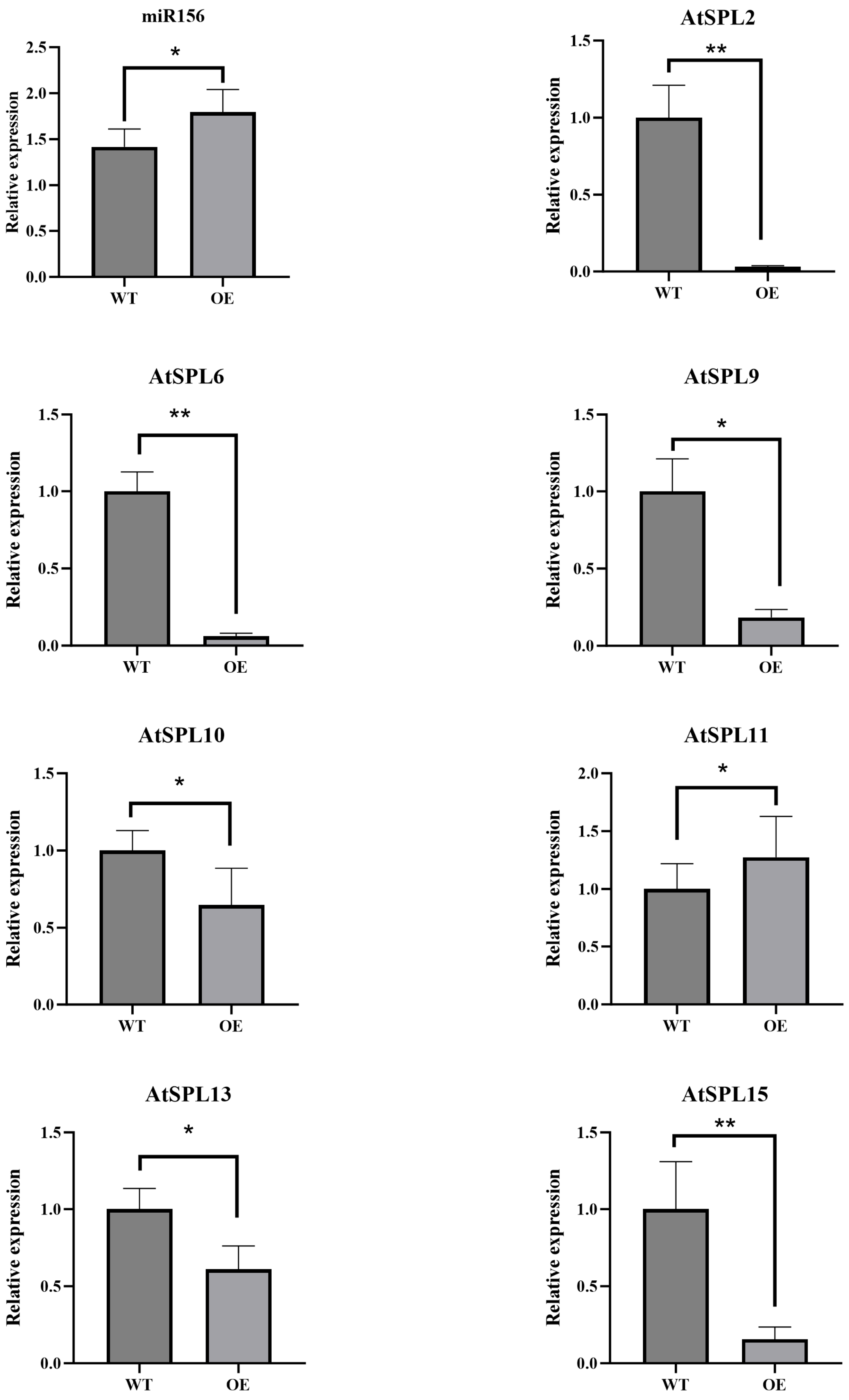

miR156
Relative expression
2.5
2.0
1.5
1.0
0.5
0.0
*
WT
OE
AtSPL2
Relative expression
1.5
1.0
0.5
0.0
**
WT
OE
AtSPL6
Relative expression
1.5
1.0
0.5
0.0
**
WT
OE
AtSPL9
Relative expression
1.5
1.0
0.5
0.0
*
WT
OE
AtSPL10
Relative expression
1.5
1.0
0.5
0.0
*
WT
OE
AtSPL11
Relative expression
2.0
1.5
1.0
0.5
0.0
*
WT
OE
AtSPL13
Relative expression
1.5
1.0
0.5
0.0
*
WT
OE
AtSPL15
Relative expression
1.5
1.0
0.5
0.0
**
WT
OE

Supplementary Fig.S4.Expression profiles of mature LkmiR156 and *AtSPLs* in *Arabidopsis thaliana*. Data are presented as mean ± SD (n = 3 biological replicates). Statistical significance was determined by t-test. (*p<0.05, **p<0.01).

Supplementary Table S1 Specific primers for *LkMIR156s* polymerase chain reaction (PCR) amplification.

Primer name	Forward primer (5'-3')	Reverse primer (5'-3')
MIR156a	ATGGCGGACTGACTTCTG	AAGGAACCTAAAGCCTGAGAT
MIR156b1	TTGTACTCAGCCGACAGAA	CCTCTAGCGGTAAATCTCAA
MIR156b2	AATGATTTAGGGTTGTCCTCAG	TGAATACGAATAGCACCAAGAC
MIR156b3	CCAAAGGGTAATAATAGCACC	ACAGGTTCCAATCACCGT

Supplementary Table S2 Specific primers for Lkpre-miR56s qRT-PCR amplification.

Primer name	Forward primer (5'-3')	Reverse primer (5'-3')
qpre-miR156a	CTTATGGCGGACTGACTTCT	TGCTATGTGTGCTCACTCTCT
qpre-miR156b2	GATTTAGGGTTGTCCTCTGC	CGCACATAAGCCGTGTTA
qpre-miR156b3	TGACAGAAGAGAGTGGGCA	CTGACAGAAAGAGGAATGAGC
LkEF1A1	GACTGTACCTGTTGGTCGTG	CCTCCAGCAGAGCTTCAT

Supplementary Table S3 Specific primers for *AtSPLs* qRT-PCR amplification.

Primer name	Forward primer (5'-3')	Reverse primer (5'-3')
qAtSPL2	ACCGCAAGAGCCGAGTTATT	GGGTAAAACGCCTTGGTTGG
qAtSPL6	CACGCTCTACTCGTGCTCTC	CCAGCAGAATTGGTCTTGCC
qAtSPL9	TCTACTTCGAGGACGGTGGT	TCCACAAACTCGGTGTCTCG
qAtSPL10	CAAGTTCCGCGTTGCCAAAT	GCACTTTGAATGCGTTTCGC
qAtSPL11	AAGTTAGCGTGAGTGGCCTG	TCGGCAGCTTCGTTTCTTCT
qAtSPL13	TTCGAGTTATGGGCAGAGCC	CACACAACGATGCGGTTCTC
qAtSPL15	CATTTCAACCCATCAGCCGC	GCTGGCTCCGAGATTTGACT
